# Dosimetric applicator characterization in hyperthermia: an automated quality assurance procedure based on relative specific absorption rate measurements

**DOI:** 10.1007/s00066-025-02489-7

**Published:** 2025-12-12

**Authors:** Timoteo Daniel Herrera, Remko Zweije, H. Petra Kok, Johannes Crezee

**Affiliations:** 1 Radiation Oncology, Amsterdam UMC location University of Amsterdam, Meibergdreef 9, 1105AZ Amsterdam, The Netherlands; 2 Treatment and quality of life, Cancer biology and immunology, Cancer Center Amsterdam, Amsterdam, The Netherlands

**Keywords:** Effective field size, Effective penetration depth, Electric field measurement, Microwave hyperthermia systems, Repeatability evaluation

## Abstract

**Purpose:**

Ensuring reproducible treatments and therapeutic temperature rises is pivotal for wider clinical application of hyperthermia. We propose a fast automated quality assurance (QA) procedure using E‑field measurements for relative specific absorption rate (SAR) applicator characterization, with a similar workflow to radiotherapy dosimetry.

**Methods:**

Our procedure was demonstrated for 434 MHz superficial contact flexible microstrip applicators (CFMA) with similar antennas and geometry to the ALBA ON 4000D system. Applicators were placed on a liquid saline phantom. A Cartesian robot performed E‑field measurements moving E‑field sensors through the phantom. We analysed the influence of spatial resolution on effective field size (EFS) measurements. Using volume measurements, we established effective penetration depth (EPD) variation across the aperture. We evaluated repeatability, measuring central planes on eight different days. Applicators/phantoms were characterized with flat and curved setups, using standard clinical and more excessive bolus thicknesses.

**Results:**

Procedures take ~40 min per setup condition. One-centimetre spatial resolution appears sufficient for QA. EPD showed errors < 5% when determined from high-SAR regions (~80–90% of the maximum). The EFS and EPD variability between different days was < 5%. Increasing EPD and decreasing EFS with increasing curvature was observed when using the clinical bolus thickness, with more homogeneous SAR distributions for curved than for flat setups. Excessive bolus thickness resulted in irregular SAR distributions and larger EFS and EPD variations.

**Conclusion:**

The proposed QA procedure is characterized by fast, practical and reproducible measurements that are suitable for efficiently evaluating various setup conditions. This flexible workflow can also be used with other radiative hyperthermia systems.

## Introduction

Mild hyperthermia, i.e., heating tumours to temperatures of approximately 40–43 °C for 1 h, is a clinically proven radio- and chemotherapy enhancer for several tumour sites [1–7]. Furthermore, a thermal dose–effect relationship has been demonstrated for some tumour sites, relating higher achieved temperatures to improved outcome [8–10] and emphasizing the relevance of achieving good control over the temperature distribution.

Superficial hyperthermia refers to the application of hyperthermia for superficial tumours (from the skin up to ~4 cm depth), where the effectiveness of combining hyperthermia and radiotherapy as well as thermal dose–effect relationships have been demonstrated for locally advanced and recurrent breast cancer [2, 6, 8, 9, 11], head and neck cancer [7], and melanoma [3, 12]. These thermal dose–effect relationships highlight the importance of delivering hyperthermia in a consistent and reproducible manner and of achieving sufficiently high power/temperature levels—in clinical trials, to allow reliable assessment of treatment outcomes, and in daily practice, to ensure that patients receive treatments of consistently high quality [13]. Such consistency in delivery facilitates interpretation of thermal dose–effect relationships, even though it does not automatically ensure achievement of higher temperatures, as these also depend on the patient-specific local anatomy and perfusion rates.

Diverse equipment meets the clinical requirements for superficial hyperthermia, including microwave antennas, infrared sources, ultrasound transducers and radiofrequency electrodes [14]. All of these deposit energy within a limited tissue volume close to the skin surface, but the heating depth and effective field size differ. The temperature rise in the treated volume is caused by absorption of the administered power, typically quantified through the specific absorption rate (SAR) distribution.

Initial and continued regular characterization of equipment is needed to ensure that the applicators operate properly according to their specifications and to detect any malfunction. The methods used to date to characterize the SAR distributions generated by hyperthermia applicators are based on E‑field or temperature rise (TR) measurements. E‑field distributions can be measured in liquid phantoms using E‑field probes. Considering that the SAR is proportional to the E‑field squared, these measurements can be quantitative, scanning a single probe [15, 16] or using a Schottky diode sheet [17]. Qualitative assessments can also be performed for a rapid check on the field distribution, using, for example, multiple probes arranged in an LED or lamp matrix [18]. For characterization of heating devices/applicators by TR measurements, solid phantoms are used. A number of thermometry probes positioned at relevant locations then provides information about the SAR pattern, since SAR is proportional to the measured temperature rise [19].

The European Society for Hyperthermic Oncology (ESHO) has developed quality assurance (QA) guidelines with technical requirements for heating devices used in superficial hyperthermia. These guidelines aim for a uniform procedure for characterization of the performance of hyperthermia applicators to make treatment delivery more reproducible. The latest ESHO guidelines propose applicator characterization based on temperature rise measured with infrared cameras and dedicated tissue-mimicking split phantoms [20]. They recommend complementing the TR characterization of the hyperthermia system with SAR experimental assessment or simulations, also in line with earlier ESHO QA guidelines [21].

The QA guidelines recommend the *effective field size* (EFS) and the *effective penetration depth *(EPD) as relevant quality metrics for SAR characterization. The EFS is “the area within the 50% of maximum SAR contour in the 1 cm deep plane” [20]. This value is often reported as the maximum extension of the 50% isoline along the main central axes of the applicator, i.e. EFS = EFS_x_ × EFS_y_. The EPD is “the depth where the SAR falls to 50% of the maximum SAR at 1 cm depth” [20]. The EFS and EPD roughly define the volume that will be reached by the 50% SAR, thus allowing appropriate applicator selection and configuration depending on the geometry of the treatment volume.

A drawback of the QA procedures described above is that they can be very time consuming. Implementation of TR-based QA requires preparation and characterization of the phantoms, which can be demanding. Furthermore, a waiting time of several hours is needed between subsequent measurements to allow the phantoms to cool down and avoid any influence of thermal conduction [22]. If thermocouple thermometry is used, the high thermal conduction along probe wires may cause smearing effects that affect the results [19, 23–25]. Due to manual positioning and repositioning of the measuring probes, SAR characterization through E‑field measurement is often time consuming (and user dependent), resulting in lengthy procedures when measuring E‑field distributions for larger applicators.

Quality assurance procedures in hyperthermia could become significantly faster and user friendlier by using similar automated scanning procedures to those applied in radiotherapy QA, where regular dosimetric characterization of linear accelerators can be performed with ionization chambers in water, measuring dose distributions with automated scanners [26, 27]. This has proven to be fast, reliable and easy to reproduce at different timepoints and for different hospitals. In this work, we propose a similar automated QA procedure for characterization of hyperthermia applicators using an automated scanner to measure the E‑field in a liquid phantom. The methodology can be generally used for radiofrequency/microwave applicators, but we demonstrate the procedure here with contact flexible microstrip applicators (CFMA) [28] operating at 434 MHz (SRPC Istok, Fryazino, Moscow region, Russia), as used for superficial hyperthermia. The advantage of using CFMAs to demonstrate the proposed procedure is their bendability; this means that these applicators can be used both flat and curved, thereby covering the wide range of curvature conditions that can occur in commonly used applicators for superficial hyperthermia, including the most commonly used commercially available applicators such as the ALBA ON 4000D (Medlogix SRL, Rome, Italy) or BSD-500 (Pyrexar Medical, Salt Lake City, UT, USA) systems or the in-house developed 434 MHz Lucite cone applicator (LCA) [29]. In particular, the design of the ALBA applicators is almost identical to that of the CFMAs, except for the fixed curvature of the ALBA applicators. We show that this method enables efficient routine QA, allowing repetitive measurements for different antennas and for relevant parameters. As an example, we illustrate an application of the methodology for varying water bolus thickness and curvature of the contact surface.

## Materials and methods

In this work, we propose a QA setup and procedure for automated E‑field scans in a liquid phantom, followed by an example application in CFMA characterization (with several curvature conditions and bolus thicknesses, thus showing that the methodology is suitable for a wide range of superficial applicators). First, we describe the measurement setup (“E-field measurement setup”) and scanning procedure (“E-field measurement procedure”), followed by the application of the QA workflow for applicator characterization with several setup conditions and analyses (Table 1), describing the methods and presenting the results (“Sensor calibration”, “Spatial resolution”, “EPD determination”, “Repeatability evaluation”, “Applicator characterization”). Applicator characterization was performed with contact flexible microstrip applicators (CFMAs) for clinical (1.3 cm) and non-clinical (2.5 cm) bolus thickness, with a PVC contact surface with the saline solution, either flat or curved with radii of 17.5 or 28 cm, in all cases matching the curvature given to the CFMA. The diversity of setup conditions demonstrates applicability of the procedure either with flat (BSD-500) or curved (ALBA) applicator systems. When implementing this QA procedure for other applicators, the curvature of the contact surface with the phantom should match the (fixed) curvature of the applicator. It is also important to evaluate the required spatial resolution, repeatability, and the criteria for EPD determination, since these aspects might be influenced by the operating frequency, effect of water bolus, applicator size, curvature and other characteristics relevant for that specific applicator type.

**Table 1:** Summary of the measurements presented in this work, with the setup conditions and analysis performed. Each measurement is described/shown in the similarly named “Materials and methods”/“Results” section

Description (section)	Applicator	Bolus thickness (cm)	Contact surface	Grid (cm)	Measured geometry	Analysis
Sensor calibration	5H	1.3	Flat	1	Centre of the applicator at 1 cm depth	Quadratic fit to convert V_out_ to SAR
Spatial resolution	3H	1.3	Flat	0.25	xy-plane	Resampled to grids of 1, 2 and 4 cm. Comparison of obtained EFS_x_, EFS_y_ and isolines
EPD determination	5H	1.3	Flat	1	Volume	∆EPD(x, y), region where ∆EPD < 5%
Repeatability evaluation	5H	1.3	Flat	1	xy-, xz-, yz-planes; eight different days	Average and SD for EFS_x_, EFS_y_, EPD, ∆_x_ and ∆_y_
Applicator characterization	3H, 5H	1.3, 2.5	Flat, curved	1	xy-, xz-planes	Comparison of EFS_x_, EFS_y_, EPD, isolines and SAR patterns

Applicator characterization was performed with contact flexible microstrip applicators (CFMA, SRPC Istok) for clinical (1.3 cm) and non-clinical (2.5 cm) bolus thickness, with a PVC contact surface with the saline solution, either flat or curved with radii of 17.5 or 28 cm, in all cases matching the curvature given to the CFMA.

$\Updelta EPD\left(x,y\right)=\frac{EPD\left(x,y\right)-EPD(\max SAR)}{EPD(\max SAR)}\times 100{\%}$, where *EPD(x, y)*, is the depth where the SAR values fell to 50% of the value at 1 cm depth for the coordinates (x, y)

*∆X, ∆Y* effective field centre shifts, difference between the geometrical centre determined from EFSX and EFSY and the origin determined visually at the centre of the applicator; *EFSX, EFSY* maximum extensions of the 50% isoline along the main central axes of the applicator; *SAR* specific absorption rate (J/kg); *SD* standard deviation; *Vout* measured voltage at the E‑field sensor; *xy-plane* surface at 1 cm depth in saline below the contact surface (flat or following the curvature; Fig. 2a shows the curved situation); *yz- and xz-planes* planes going through the centre of the applicator along the X and Y axes, respectively, scanned from 1 cm depth up to an additional ~5 cm depth

We describe the sensor calibration to obtain relative SAR distributions from E‑field measurements (“Sensor calibration” sections in “Materials and Methods” and “Results”). Then we analyse the influence of spatial resolution, considering its impact on EFS determination and the obtained qualitative SAR distribution (“Spatial resolution evaluation” sections in “Materials and Methods” and “Results”). We also evaluate the error in estimating EPD from the planes going through the centre of the applicator instead of in the position of maximum SAR (measuring said planes increases automation of the QA procedure; “EPD determination” sections in “Materials and Methods” and “Results”). We determine the repeatability of EFS and EPD determination as well as the applicator positioning accuracy when performing several measurements with the same setup on different days (“Repeatability evaluation” sections in “Materials and Methods” and “Results”). We show as an example the SAR characterization of CFMAs for several curvature conditions and with clinical and non-clinical bolus thickness (“Applicator characterization” sections in “Materials and Methods” and “Results”). Finally, we summarize the implementation of the QA procedure, taking into account the previous sections (“QA procedure implementation” and “Discussion”).

### E-field measurement setup

**Fig. 1:**
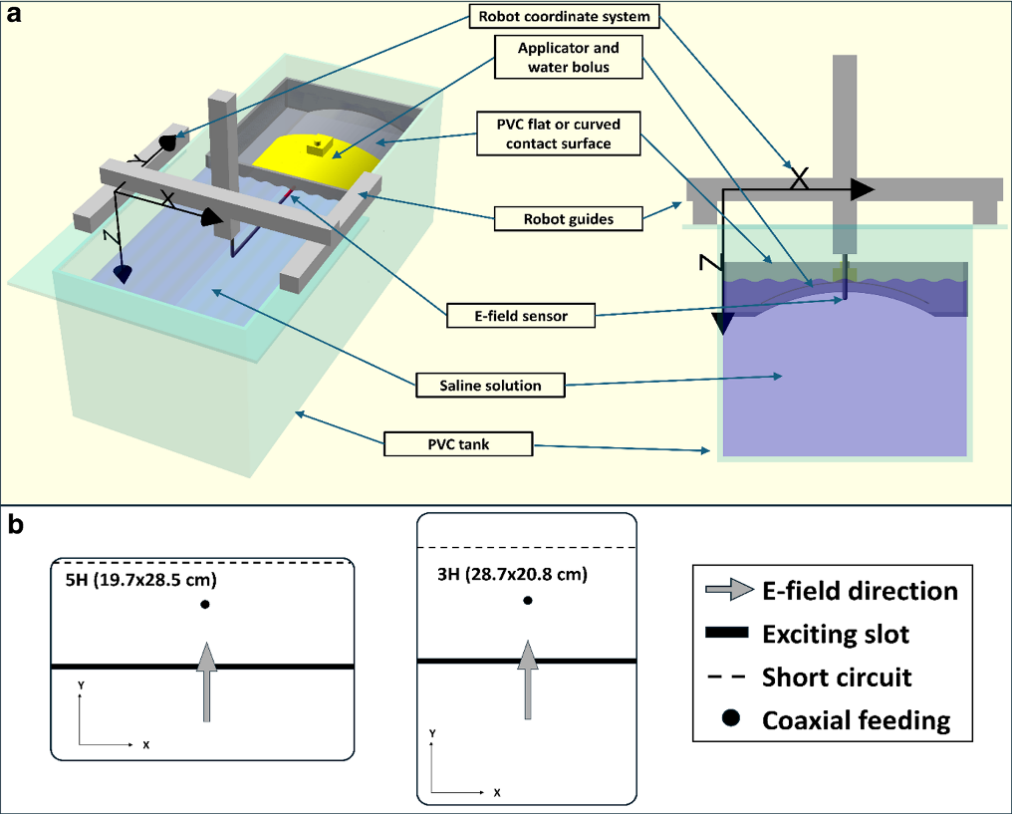
**a** Schematic tridimensional (left) and frontal view (right) representations of the experimental setup for automated E‑field measurements for specific absorption rate (SAR) characterization of superficial hyperthermia applicators. The sensor is moved by a Cartesian robot allowing fast and accurate measurements of any volume or surface. **b** Schematic representation of the contact flexible microstrip applicators (CFMA, SRPC Istok) 5H and 3H, also showing their positioning relative to the robot coordinate system

Contact flexible microstrip applicators have an integrated water bolus for coupling of the applied power and circulation of heated water and can be bent to adapt to the curvature of the patient surface in the treatment area. In previous studies the performance of CFMAs has been extensively characterized through simulations and measurements [15, 28, 30–32]. In particular, these studies showed a larger influence of curvature [31] and resonance effects for larger bolus thicknesses [32] for the two CFMAs with the largest aperture size—3H (28.7 × 20.8 cm^2^) and 5H (19.7 × 28.5 cm^2^)—as well as more challenges to ensuring reproducibility for repeated measurements [30]. We therefore focused the evaluation of our proposed QA workflow on these two applicators. Furthermore, the 3H applicator has the same geometry as the ALBA Delta applicator (with an aperture size of 30 × 22 cm^2^), except that the Delta has a fixed curvature of 17.35 cm. Figure 1a shows a schematic view of the E‑field measurement setup, with a representation of the 3H and 5H geometry in Fig. 1b.

We performed E‑field measurements in a PVC tank of 42 × 100 × 40 cm^3^ filled with saline solution (3 g/L NaCl). At a frequency of 434 MHz and room temperature of ~21 °C, the electrical conductivity of the phantom was σ = 0.53 S m^−1^ and the permittivity ε = 78.7 [33]. We checked the electrical conductivity of the phantom before every measurement using an EC meter (DeltaOhm HD2106.2, Senseca Italy Srl, Caselle di Selvazzano, Italy), verifying that the measured values corresponded to the desired concentration of saline solution. Applicators were connected to an ALBA ON 4000D system (Medlogix Srl, Rome, Italy) signal generator operating at 434 MHz. A PVC contact surface was positioned over the tank, either flat with a thickness of 2 mm or with curvature radii of 17.5 cm and 28 cm, with a thickness of 1 mm. For every measurement, the curvature of the PVC contact surface matched the curvature given to the bendable CFMA. No fat layer was used. A thin layer of 1 mm polyethylene foam (IPS Karton.eu GmbH & Co., Spremberg, Germany) was introduced between the metal applicator and the water bolus to improve the repeatability of the air distribution in the contact between the applicators and the water bolus and thus greatly reduce the impact of an irregular air distribution on the SAR distribution [30].

The applicators were centred visually using marks drawn on the contact surface. The water bolus was filled with demineralized water without circulation. To ensure uniformity of the bolus thickness, we used 3D-printed holders that were attached to the applicators, with interchangeable sticks to indicate the corresponding bolus thickness. Additional weights were placed on the applicator, ensuring uniform bolus filling and improving contact.

We used an in-house built sensor made from a 1N4148 diode dipole, with high resistance leads (~90 kΩ/m) and a total length of 2 cm. The sensor was made waterproof by submerging it in a silicon solution (Eurosil 10 orange, SynTech, Netherlands). The probe was positioned along the main E‑field direction of the applicator (see the “Sensor calibration” sections in “Materials and Methods” and “Results” regarding the calibration of the sensor) and attached to the frame of a Cartesian robot with a workspace of 500 × 500 × 24 mm (Igus Drylin Portal DLE-RG-500 × 500 × 240, Igus GmBH, Cologne, Germany). This allowed us to measure any desired surface or volume, point by point, with a positioning accuracy (reported by the manufacturer) of less than 1 mm. A GW Instek GW8342 (Good Will Instrument Co., Ltd., Taipei, Taiwan) multimetre was used to obtain a voltage reading from the sensor at each position. For each step, after the target position had been reached, there was a waiting time of 0.8 s (for stabilization of the multimetre reading) before performing three voltage measurements, of which the average was used (the variation between these readings was less than 1%).

The measurement setup (PVC tank, signal generator, diode sensor, Cartesian robot, multimetre) was assembled in house. A similar design, based in our in-house implementation, will be commercially available from Medlogix Srl (Rome, Italy).

### E-field measurement procedure

**Fig. 2:**
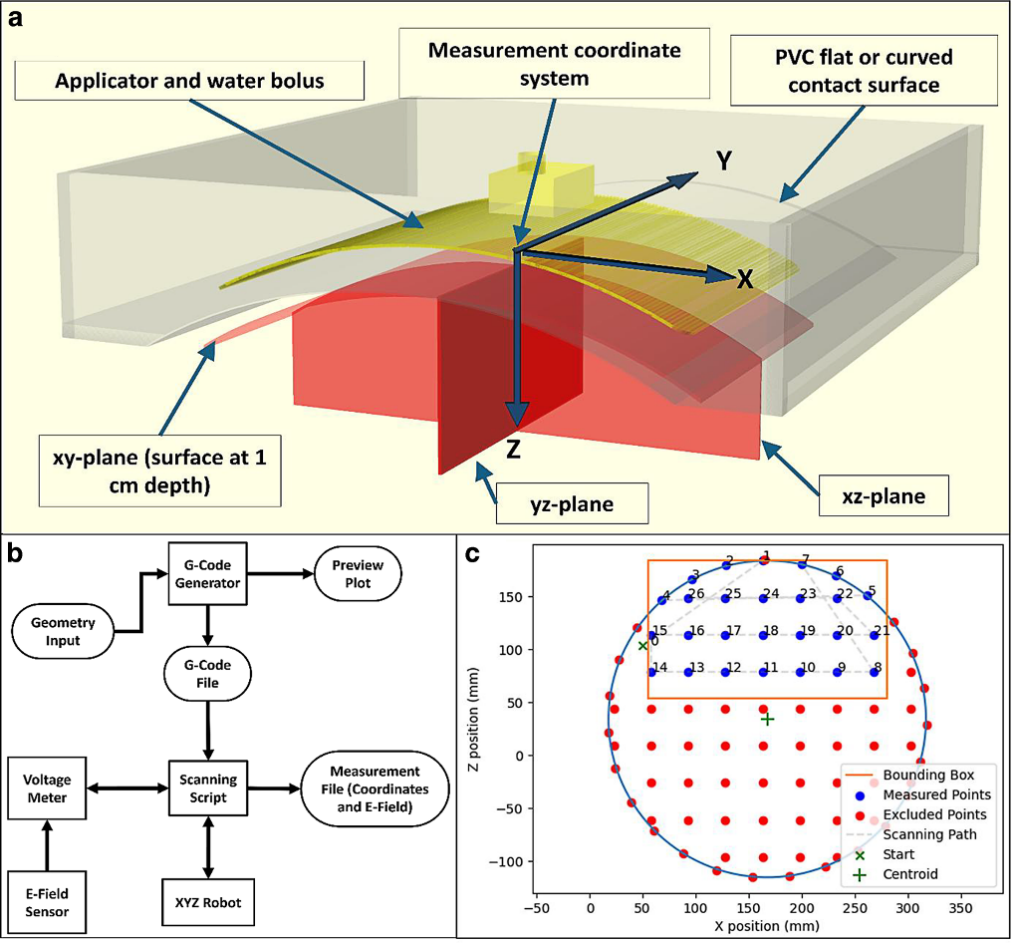
**a** Schematic representation of the measured planes with respect to the setup shown in Fig. 1. The image shows the case where the applicator and the PVC contact surface are curved. We measured with a flat setup and with curvature radii (of the contact surface) of 17.5 and 28 cm. In all cases, the curvature of the applicator and the PCV contact surface were the same. **b** Schematic workflow for automated E‑Field measurement. **c** Preview scanning plot created by the in-house developed G‑code generator. The three points to determine the 1 cm depth curve as well as the limits for the bounding box are defined by the user. The G‑code generator then creates the scanning path and the preview scanning plot. This case corresponds to curved setup, measuring the xz-plane. The circle defined is determined by three points in robot coordinates at 1 cm depth under the contact surface, and the bounding box is determined according to the depth and X extension of the measurement. The scanning path goes first through points on the edge of the circle within the bounding box and then completes the plane going through the other points inside of the bounding box according to the spatial resolution chosen for the measurement. Note that the coordinate system shown here represents a general fixed robot coordinate system. When analysing results in terms of specific absorption rate (SAR) distributions, a conversion is made such that the origin is always at the centre of the applicator (at the bolus–phantom interface), enabling easier interpretation

Figure 2 illustrates the measurement procedure, with Fig. 2a showing the measurement coordinate system and measured planes, Fig. 2b showing the schematic workflow for automated E‑field measurement, and Fig. 2c the preview scanning plot showing the geometry and scanning path.

The origin of the measurement coordinate system (Fig. 2a) was the centre of the applicator projected onto the interface between the PVC contact surface and the saline solution. The directions of the measurement axes match with the robot coordinate system axes; however, the origin of the latter is determined by the scanning limits (Fig. 1a). The measurement coordinate system matches the IEC 61217 fixed reference system of common use in radiotherapy QA [34, 35], with the “gantry” being the coaxial feeding of the applicator, reversing the direction of the Z axis to be positive with increasing depth. Aiming for a fast and smooth procedure, we scanned the main central planes going through the centre of the applicator (Fig. 2a). In this way, several bolus thicknesses and applicators could be measured with the same scanning geometry.

For EFS characterization, we measured the surface at 1 cm depth following the curvature, which we refer to as the *xy-plane *in curved and flat cases. For EPD characterization, we measured the *xz-plane *from 1 cm depth following the curvature (since EPD considers the additional depth from 1 cm [20]), up to a minimum additional depth of ~5 cm. The* yz-plane *could be used along with the xz-plane for EPD characterization. In order to evaluate the differences between the EPD determined at the position of the maximum SAR with that obtained from the xz- or yz-plane, we also performed a 3D scan, further described in the “EPD determination” sections in “Materials and Methods” and “Results”.

A Python script was used to generate a file with G‑codes to control the movement of the scanner, along with instructions for voltage reading and easy creation and visualization of the scanning planes.

The E‑field sensor was positioned visually at the centre of the applicator using manual G‑code commands. In a similar way, the robot coordinates for the scanning limits were defined by taking into account the dimensions of the applicator (bounding box shown in Fig. 2c). When measuring with a curved applicator (and matching curvature for the PVC contact surface), the robot coordinates for three points at 1 cm depth below the contact surface were located, with which the G‑code generator determined the (virtual) circle whose circumference contained the 1 cm depth curve at the centre of the applicator (see circle and centroid in Fig. 2c). The G‑code generator then determined the scanning path and robot coordinates of the points to measure for each measurement plane, considering a convenient starting point (no collisions), the scanning limits (bounding box) and the 1 cm depth surface (extending the virtual circle along the Y axis for curved setup). For example, when measuring the xz-plane, the scanning path would measure first the points at 1 cm depth (following the curvature) and then the remaining points inside of the bounding box according to the chosen spatial resolution (numbered points in Fig. 2c). The G‑code generator showed the user a preview of the scanning path and measured points (Fig. 2c shows the preview of an xz-plane for the curved setup). The created G‑code file could be used for multiple scans, provided that curvature and scan limits remain unchanged.

The scanning itself was performed with another Python script that sent the G‑code commands to the robot and the measurement commands to the voltage meter, storing the coordinates and measured voltage in a file.

### Sensor calibration

Specific absorption rate values can be derived from E‑field values using the following relation:

1 $$SAR\left(x,y,z\right)=\frac{\sigma }{2\rho }\left|\left|\overrightarrow{E\left(x,y,z\right)}\right|\right|^{2}\,\left(Wkg^{-1}\right),$$

where σ (S m^−1^) is the electrical conductivity of the medium and ρ (kg m^−3^) the density of the material. If a certain power *P* is applied, $\left|\left|\vec{E}\right|\right|^{2}$ and therefore the SAR in the phantom will be proportional to *P*.

We applied a relative calibration for the response of the sensor to obtain a correct relative SAR value from the voltage reading values *V*_*out*_ obtained with the probe. To this end, the sensor was placed at a high-SAR position at 1 cm depth in the tank, with the 5H applicator and a 1.3 cm bolus thickness, to ensure that the calibration covers the full range of V_out_ values that will be encountered. Then values of *P* between 0 and 50 W were generated, within the clinically used range for the CFMAs, measuring the corresponding *V*_*out*_. The obtained values for *V*_*out*_ were fitted using a second-order polynomial (taking into account the quadratic relationship shown in Eq. 1):

2 $$P=A*{V_{out}}^{2}+B\times V_{out}+C$$

The coefficients A, B and C were determined by nonlinear least squares fitting using the SciPy library [36]. The relative SAR was obtained by applying Eq. 2 to the measured *V*_*out*_ and normalizing to the maximum value at 1 cm depth for every scan to 100%.

For the scanned E‑field distributions in the rest of this work, a new calibration of *V*_*out*_ to P (and then to relative SAR) was made for each measurement day. Then the scans were performed with a generated power of 30 W (within the range of power values used in hospitals). The temperature and salinity were checked several times when performing extended measurements and did not show significant deviation. Relative SAR values at the measurement grid were linearly interpolated using *griddata *from SciPy [36] to a grid of 0.1 × 0.1 cm^2^ for EFS_x_, EFS_y_ and EPD determination. The figures in this work showing measured planes display the interpolated grid in colour mesh, with an indication of the measurement locations as black dots.

### Spatial resolution evaluation

The automated scans allow for high spatial resolutions of up to 1 mm, but such high resolutions require a longer scanning time. To evaluate the influence of spatial resolution on characterization, we measured the xy-plane for the 3H applicator with 1.3 cm bolus thickness and flat setup, with a grid size of 0.25 cm (smaller resolutions were not scanned due to longer scanning times). We then compared the SAR patterns, EFS_x_, EFS_y_ and isolines obtained with only taking the measured points corresponding to spatial resolutions of 1, 2 and 4 cm to the reference situation with spatial resolution of 0.25 cm.

### EPD determination

The QA guidelines define EPD as “the depth where the SAR falls to 50% of the maximum SAR at 1 cm depth” [20], thereby explicitly mentioning that “the maximum SAR may not be in the plane through the main central axes of the applicator”. Measuring the EPD according to the guideline definition would thus require us to first scan the xy-plane and then scan in depth at the position (x, y) of the maximum SAR in the xy-plane. It would also be possible to generate new G‑codes for planes parallel to the xz- and yz-planes passing by the maximum SAR position. When performing automated scans, this procedure can become time consuming, especially when several setup conditions or applicators are evaluated. On the other hand, measuring the xz- or yz-planes can be easily automated for several setup conditions and applicators. If the EPD is obtained from these planes, the result could differ from the guideline definition.

To quantify the accuracy in EPD determination when measuring the central planes of an applicator, we performed a volume measurement of the SAR distribution: for each (x, y) coordinate in the plane at 1 cm depth, we measured every 1 cm up to an additional depth of 5 cm. We used the 5H applicator with a bolus of 1.3 cm.

We calculated EPD(x, y) as the depth where the SAR values fell to 50% of the value at 1 cm depth for the coordinates (x, y). We calculated the relative difference

3 $$\Updelta EPD\left(x,y\right)=\frac{EPD\left(x,y\right)-EPD(\max SAR)}{EPD(\max SAR)}\times 100{\%},$$

where *EPD(max SAR)* is the EPD at the position of the maximum SAR at 1 cm depth, i.e., according to the guideline definition.

We evaluated the region in the xy-plane where ∆EPD was smaller than 5%. Then we evaluated whether the yz- and xz-planes passed through that region. If this was the case, then using the yz- and xz-planes gives a good estimation of EPD, allowing us to further automate the scans.

### Repeatability evaluation

We evaluated the repeatability of the obtained EFS and EPD by performing eight measurements on different days during a period of 2 weeks. Each day the applicator was repositioned; the sensor calibrated; and a scan was performed for the xy-, xz- and yz-planes. We used the 5H applicator with 1.3 cm bolus thickness and flat setup. We evaluated the average and standard deviation for the set of scans of EFS_x_, EFS_Y_, and EPD determined with the xz- and yz-planes. The standard deviations reflect the repeatability for characterization of this applicator.

For the CFMAs, differences in the observed SAR patterns between scans with the same setup are expected according to previous studies, especially for the flat setup [30–32]. This effect was reduced by using a thin foam layer between the applicator metal and the water bolus, thus regularizing the air distribution [30].

In order to estimate the positioning error, we calculated the centre shifts *∆*_*x*_ and *∆*_*y*_. The values of *∆*_*x*_ and *∆*_*y*_ are the difference between the centre of the EFS and the positioning centre. The centre of the EFS was determined as the middle point between the extreme values for the 50% SAR isoline for each scan. The positioning centre is the origin of the measurement coordinate system (Fig. 2a), which is determined visually when setting up the scans. Then *∆*_*x*_ and *∆*_*y*_ give an estimation of the positioning error for each scan (as well as due to asymmetries in the 50% isoline). On the other hand, the standard deviations of *∆*_*x*_ and *∆*_*y*_ give the positioning uncertainty for each axis.

### Applicator characterization

To show the application of the proposed QA procedure for applicator characterization, we measured the xy- and xz-planes for the 3H and 5H applicators. We used bolus thicknesses of 1.3 cm (close to clinical practice usage) and 2.5 cm (a thicker bolus where resonance effects could be expected [32]). Routine QA measurement should be sufficient with a homogeneous bolus thickness close to the thickness commonly used in clinical practice. However, we included an additional measurement with a thicker bolus to illustrate possible problems that might arise when using the wrong bolus thickness.

We calculated EFS_x_ and EFS_y_ from the xy-plane. The EPD was calculated using the maximum SAR at the xz-plane. We also evaluated the 70%, 80%, 90% and 98% isolines and SAR patterns for each setup condition.

### QA procedure implementation

A summary of the measurements and analyses described in the previous sections can be found in Table 1.

**Fig. 3:**
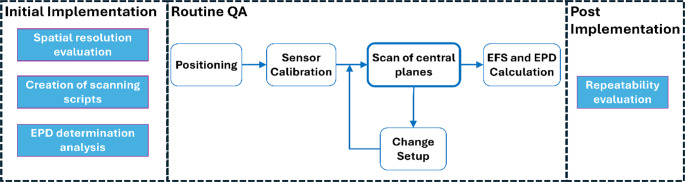
Workflow for the implementation of the QA procedure for automated SAR-based dosimetric characterization of hyperthermia applicators. The steps in full rectangles are required for implementation, since some relevant parameters for measurement or evaluation are obtained from them. The steps performed for routine QA measurements are in rounded rectangular outlines. *EFS* effective field size, *EPD* effective penetration depth

Figure 3 shows the workflow for the implementation of the QA procedure, taking into account the considerations and measurements described in the previous sections (see also Table 1). This workflow is general and can be used for any radiative hyperthermia system. The creation of scanning scripts should consider the applicator and setup geometry (Sect. “E-field measurement procedure” of “Materials and methods”). The spatial resolution evaluation and EPD determination analysis establish additional considerations for how the routine QA measurements should be performed (Sects. “Spatial resolution evaluation” and “EPD determination” of “Materials and methods”). Then routine QA can be performed for the setup conditions to be evaluated (Sect. “Applicator Characterization” of “Materials and methods”), including a previous calibration of the sensor (Sect. “Sensor calibration” of “Materials and methods”). The expected variability in the results of several routine QA measurements can be assessed with a repeatability evaluation (Sect. “Repeatability evaluation” of “Materials and methods”).

## Results

### Sensor calibration

**Fig. 4:**
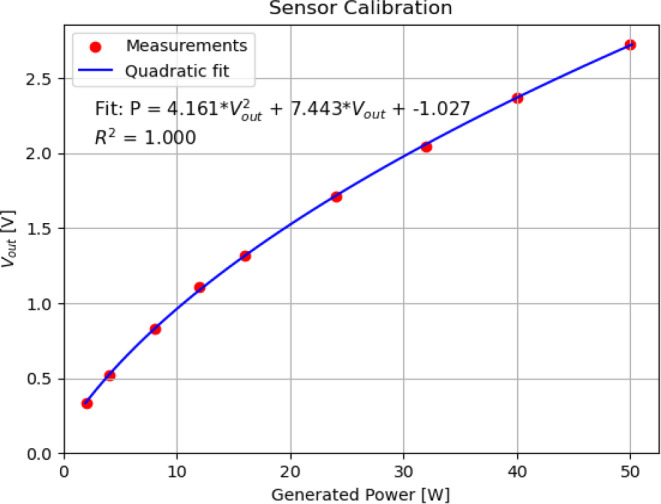
Calibration of the response of the E‑field sensor to generated power. The sensor was placed at 1 cm depth, at the centre of the 5H applicator, with 1.3 cm bolus and flat setup. Power was generated with an ALBA ON 4000D 434 MHz signal generator, recording the measured voltage at the sensor, *V*_*out*_. Power was fit as a quadratic function of *V*_*out*_ according to Eq. 2. The parameters of the fit as well as the values for R^2^ are shown in the figure

Figure 4 shows the results of calibration of the sensor to relative SAR values. The calibration was performed using the 5H applicator with 1.3 cm bolus in the flat setup. Figure 4 also shows the obtained values for the quadratic fit according to Eq. 2 as well as the value for R^2^.

For this calibration, the sensor was positioned at the centre of the applicator at 1 cm depth, corresponding to a high SAR value close to the maximum of the distribution. This ensures that the calibration covers the full range of *V*_*out*_ values that will be measured.

### Spatial resolution evaluation

**Fig. 5:**
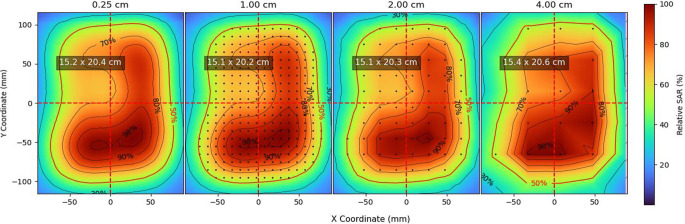
Spatial resolution evaluation. The specific absorption rate (SAR) distribution at 1 cm depth was measured for the 3H applicator with 1.3 cm bolus and flat setup using a separation between measurements of 0.25 cm. The distribution was then resampled to mimic spatial resolutions of 1, 2 and 4 cm. All distributions were then interpolated to a 0.1 cm grid, from which the 50% contour was determined as well as the effective field sizes along the X and Y axes, EFS_x_ × EFS_y_ (shown for every spatial resolution). The black dots show the measurement positions (omitted for the 0.25 cm resolution)

Figure 5 shows the results of the spatial resolution evaluation. The xy-plane SAR distribution obtained with a grid resolution of 0.25 cm was resampled to spatial resolutions of 1, 2 and 4 cm (no interpolation was performed; in each case, only some of the measured points were included, which are shown in Fig. 5 by black dots). The obtained values for EFS_x_ × EFS_y_ were 15.2 × 20.4 cm, 15.1 × 20.2 cm, 15.1 × 20.3 cm and 15.4 × 20.6 cm for spatial resolutions of 0.25, 1, 2 and 4 cm, respectively, in all cases calculated with an interpolated grid of 0.1 × 0.1 cm^2^. The differences in the obtained field sizes with reduced spatial resolution are less than 2%.

The SAR patterns and isolines do not show major differences for the resolution of 1 cm with respect to that of 0.25 cm, which makes 1 cm an appropriate resolution for capturing the qualitative aspects of the SAR distribution. However, by reducing the spatial resolution to 2 or 4 cm, some aspects of the SAR patterns are lost. Especially the higher isolines of 98 and 90% change for the resolutions of 2 and 4 cm. The resolution of 4 cm also shows differences for the 70 and 80% isolines.

An xy-plane scan of the 3H applicator with a spatial resolution of 1 cm took ~20 min, with a total of ~40 min when adding the xz- and yz-planes.

### EPD determination

**Fig. 6:**
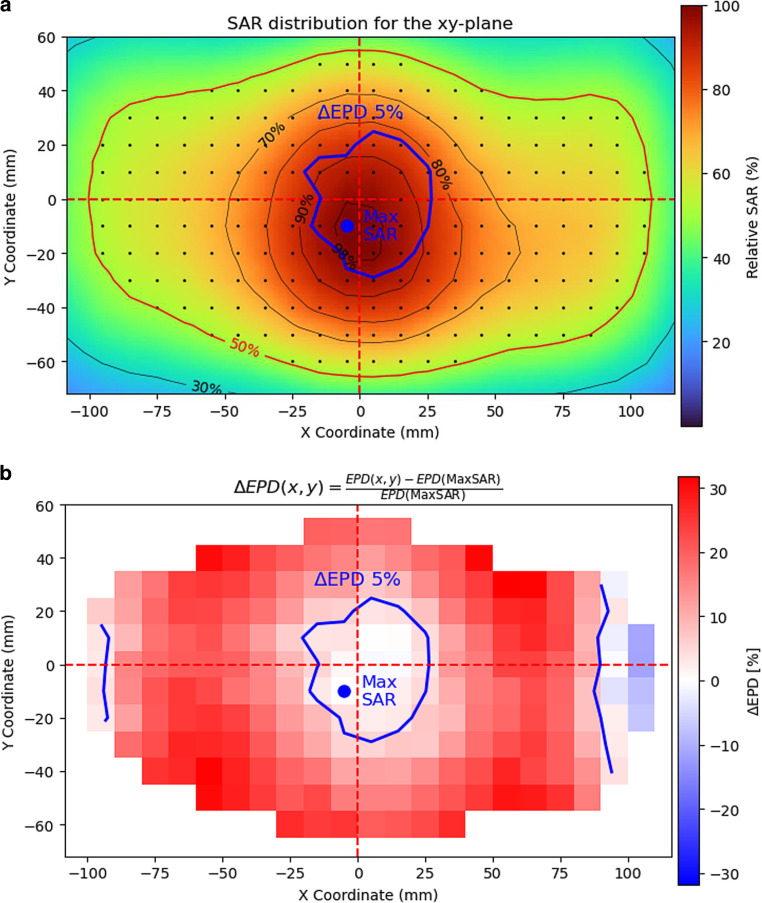
Error in effective penetration depth (EPD) determination across the xy-plane, with a 3D measurement of the 5H applicator, 1.3 cm bolus and flat setup: **a** xy-plane, showing also the relative isolines and the measured positions (dots); **b** Relative EPD differences $\Updelta EPD\left(x,y\right)=\frac{EPD\left(x,y\right)-EPD(\max SAR)}{EPD(\max SAR)}\times 100{\%}$, where *EPD(x, y)*, is the depth where the specific absorption rate (SAR) values fell to 50% of the value at 1 cm depth for the coordinates (x, y). In **a **and **b**, the coordinate with the maximum SAR is indicated, as is the contour that encloses the coordinate region where ∆EPD is lower than 5%. **b** shows that the X and Y axes go through the region where ∆EPD < 5%. Therefore, EPD determination by measuring only the central planes (instead of the planes going through the maximum SAR value) gives an error below 5%, acceptable for routine quality assurance

Figure 6 shows the results of a volume measurement. For each (x, y) coordinate in the plane at 1 cm depth, we measured every 1 cm up to an additional depth of 5 cm, performed with the 5H applicator with 1.3 cm bolus and flat setup. We performed this measurement to quantify the error in EPD determination when measuring the central planes of an applicator instead of the planes going through the maximum SAR (this is more practical when performing measurement for several setup conditions or applicators). Figure 6a shows the xy-plane, with the measured points (x, y), indicating the maximum SAR and the 50% isoline. Figure 6b shows $\Updelta EPD\left(x,y\right)=\frac{EPD\left(x,y\right)-EPD(\max SAR)}{EPD(\max SAR)}\times 100{\%}$, where *EPD(x, y)* is the depth where the SAR values fell to 50% of the value at 1 cm depth for the coordinates (x, y). The contour enclosing the region where ∆EPD is lower than 5% is highlighted in both figure parts.

The EPD value at the maximum SAR was 2.6 cm, so within the enclosed area in Fig. 6, EPD differences are less than 0.13 cm. Both the X and Y axes (red dashed lines) go through the region where ∆EPD < 5%. Therefore, the EPD obtained from the xz- or yz-planes for this measurement yields a difference from the guideline definition of EPD that is lower than 5%.

Comparing both parts of Fig. 6 shows that the values of ∆EPD are lower in regions with high relative SAR. When the X and Y axes pass through the high-SAR region in the xy-plane (> 80–90% isolines), the error in EPD estimation using the xz- or yz-planes is minimal, thus not requiring additional measurements at the position of the maximum SAR. In case the high isolines for the xy-plane are far from the central X and Y axes, an additional measurement should be made at the position of the maximum SAR, in order to obtain an accurate estimation of EPD.

### Repeatability evaluation

Table 2 shows the averages and standard deviations (SD) of the QA parameters obtained from scans performed on eight different days for the 5H applicator with 1.3 cm bolus thickness and flat setup. The variability in EFS_x_, EFS_y_ and EPD was below 5%, with no significant difference in EPD determination by using the xz- or the yz-plane. The average values of *∆*_*x*_ and *∆*_*y*_ show that the effective centre of the applicator is displaced ~1 cm in each axis with respect to the geometric centre. The positioning uncertainty (SD of *∆*_*x*_ and *∆*_*y*_) is ~0.5 cm, mostly due to visual misalignment, but it sufficiently accurate for QA and EFS/EPD determination.

**Table 2:** Repeatability: eight measurements were performed on different days with the 5H applicator, 1.3 cm bolus, flat setup.

5H applicator, flat setup, 1.3 cm bolus	Average [cm]	SD [cm] (%)
EFS_x_ (along the slot)	20.9	1.0 (5%)
EFS_y_ (across the slot)	12.5	0.6 (5%)
*∆*_*x*_ (centre shift along the slot)	0.8	0.4
*∆*_*y*_ (centre shift across the slot)	−0.9	0.6
EPD with the xz-plane	2.74	0.11 (4%)
EPD with the yz-plane	2.71	0.15 (5%)

Effective field sizes along the X and Y axis, EFS_x_ and EFS_Y_, are calculated using the maximum and minimum coordinate values for the 50% contour of the maximum relative specific absorption rate (SAR) for each measurement. ∆_x_ and ∆_y_ are the offset coordinates of the centre of the 50% contour, with respect to the origin of the measurement coordinates, which was aligned visually with the centre of the applicator. Effective penetration depth (EPD) was calculated for the xz- and yz-planes from the maximum SAR at each plane. SD: standard deviation.

### Applicator characterization

**Fig. 7:**
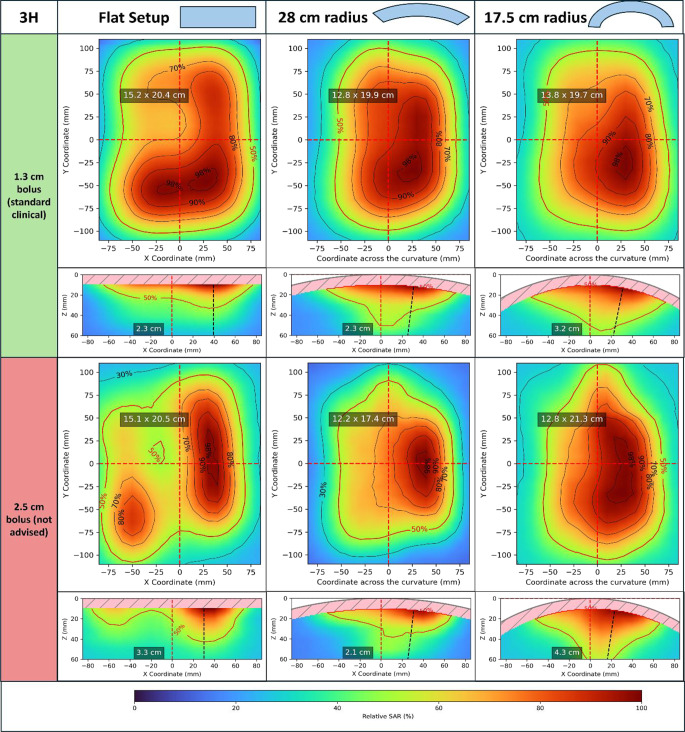
3H applicator characterization. Relative SAR distributions for flat setup and with curvature radii of 28 and 17.5 cm (see the schematic of the measurement coordinate system and measurement planes in Fig. 2). Two bolus thicknesses were used: 1.3 cm, close to clinical use, and 2.5 cm, not recommended for clinical use. For each setup and bolus thickness, the xy-plane SAR distribution is shown above, indicating in the plot EFS_x_ × EFS_y_. Below, the xz-plane SAR distribution can be seen (notice that the measurement starts at 1 cm depth, following the curvature). In the xz-plane, the EPD determined from that plane is shown, as is the line along which it was determined, starting from the maximum SAR at that plane (dashed black line). *EFS*_*X*_, *EFS*_*Y*_ maximum extensions of the 50% isoline along the main central axes of the applicator; *EPD* depth where 50% of the maximum SAR is reached, at the position of the maximum SAR of the measured plane; *SAR* specific absorption rate

**Fig. 8:**
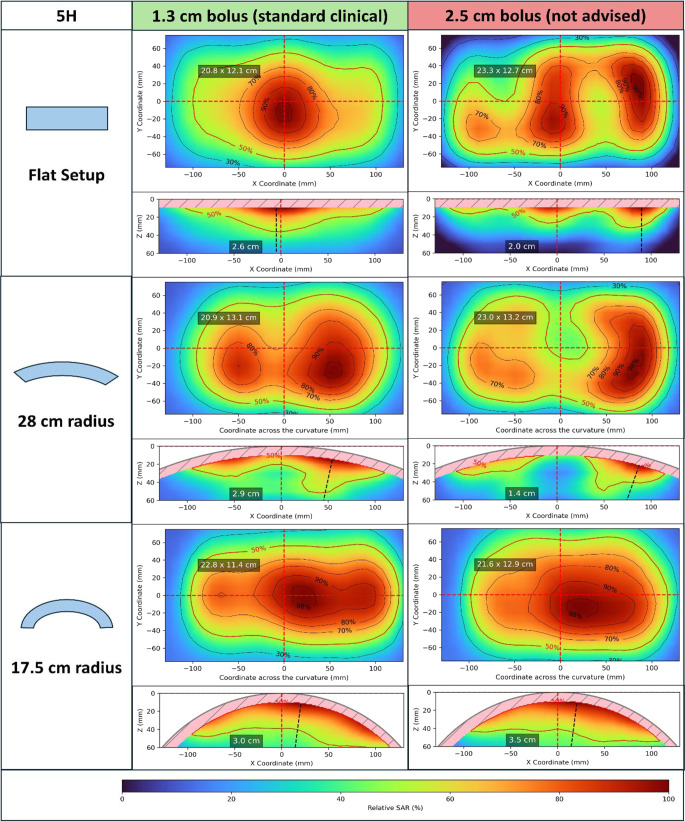
5H applicator characterization. Relative SAR distributions for flat setup and with curvature radii of 28 and 17.5 cm (see the schematic of the measurement coordinate system and measurement planes in Fig. 2). Two bolus thicknesses were used: 1.3 cm, close to clinical use, and 2.5 cm, not recommended for clinical use. For each setup and bolus thickness, the xy-plane SAR distribution is shown above, indicating in the plot EFS_x_ × EFS_y_. Below, the xz-plane SAR distribution can be seen (notice that the measurement starts at 1 cm depth, following the curvature). In the xz-plane, the EPD determined from that plane is shown, as is the line along which it was determined, starting from the maximum SAR at that plane (dashed black line). *EFS*_*X*_, *EFS*_*Y*_ maximum extensions of the 50% isoline along the main central axes of the applicator; *EPD* depth where 50% of the maximum SAR is reached, at the position of the maximum SAR of the measured plane; *SAR* specific absorption rate

Figures 7 and 8 show the application of our procedure for characterization of the 3H and 5H applicators, respectively. The plots show the relative SAR distributions for said applicators with flat setup and curvature radii of 28 and 17.5 cm. Planes with a bolus thickness of 1.3 cm, close to standard clinical use, are highlighted in green. We also show the results for a thicker bolus of 2.5 cm to highlight the importance of appropriate bolus thickness in QA and clinical use for avoiding resonance effects [32]. This second bolus thickness is highlighted in red in Figs. 7 and 8.

For each setup and bolus thickness, the xy-plane is shown, indicating EFS_x_ × EFS_y_. The xz-plane is also displayed, as is the EPD determined from that plane. The line along which EPD was determined, starting from the maximum SAR at that plane, is plotted as a black dashed line. Only the xz-plane is shown, since it also shows the SAR variation along the curvature. However, for EPD determination, the yz-plane could also be used.

For the clinical bolus thickness of 1.3 cm, there is a slight increase in EPD with curvature. The SAR patterns for both applicators also become more homogeneous with curvature, which can be seen as a larger extension for the higher isolines. Larger changes in field size are seen along the curvature (EFS_x_), which is expected due to the bending of the applicator. The changes in field size in the non-curved direction (EFS_y_) are within the expected variability observed in Table 2.

The non-clinical bolus thickness of 2.5 cm, on the other hand, shows more heterogeneous SAR distributions, which are characterized by the splitting of the 50% and higher isoline regions in some cases. This increased heterogeneity is reflected in a less clear effect on EPD, with the xz-plane showing very irregular distributions in some cases. The irregularities caused in the 50% isoline of the xy-plane by the thicker bolus result in higher variation in EFS_x_ and EFS_y_ between setup conditions than with the clinical bolus thickness.

In all the measured setup conditions, the Y axis in the xy-plane goes through high-SAR regions (it crosses the 98 and 90% isolines in most cases, the 80% isoline in some). Following the analysis of Sect. “EPD determination” of the “Results”, the EPD calculated from the xz-plane provides a good estimate of the EPD calculated from the maximum SAR at 1 cm depth. This approach eliminates the need for manual repositioning between scans.

The analysis of the whole set of measurements for applicator characterization provides valuable insights for clinical application. As shown in Figs. 7 and 8, excessively thick boluses should be avoided, aligning with previous observations [32]. Additionally, the SAR distributions (extension of higher-percentage isolines) highlight the advantage of curved applicators in achieving more homogeneous SAR coverage, consistent with earlier findings [31].

## Discussion

This work presented a fast (~ 40 min per setup condition) and effective SAR-based dosimetric characterization procedure for QA of hyperthermia applicators using automated E‑field measurements in a saline solution phantom. The procedure (Fig. 3) was demonstrated with the 3H and 5H CFMAs (SRPC Istok, Fryazino, Moscow region, Russia), but its use can be extended to a diversity of EM-based applicators and hyperthermia systems, not only for superficial hyperthermia, but also for devices used for locoregional heating.

An xy-plane scan of the 3H applicator with a spatial resolution of 1 cm took ~20 min, with a total of ~40 min when adding the xz- and yz-planes. Increasing the resolution to 0.25 cm means a ~16-fold time increase, with no significant additional information on the SAR distributions. A spatial resolution of 1 cm for the E‑field scans results in fast and practical measurements and gives enough information for detailed applicator characterization.

Spatial resolutions of 2 and 4 cm reduce the time by a factor of ~4 and ~16 with respect to the spatial resolution of 1 cm, respectively (the reduction factors might be smaller because the displacement time between measurement positions becomes more relevant). Even though some details of the SAR patterns are not captured with these spatial resolutions, the obtained EFS is very similar, and the overall shape of the isolines is kept with respect to the resolution of 1 cm. These resolution values can be used for routine QA and comparison with reference SAR distributions measured with a resolution of 1 cm, or to detect malfunctioning with a fast scan.

Using a 3D measurement, we computed the differences in EPD according to the position in the aperture xy-plane where it is determined. The conclusion of this analysis was that a good estimation of the EPD (error below 5%) is obtained from the measured xz- or yz-planes when they pass through high-SAR regions (~80–90%) in the xy-plane. We evaluated repeatability with measurements of the central planes on eight different days. Variability in EFS and EPD was less than 5%, and positioning uncertainty was below 0.5 cm. We characterized both the 3H and the 5H applicators for flat and two curved setups, both with a standard clinical bolus thickness and an excessive thickness. With the standard bolus thickness, increasing curvature of the setup showed increasing EPD and decreasing EFS. The SAR distributions for curved setups were more homogeneous than for flat setups. On the other hand, excessive bolus thickness was associated with irregularities in the SAR isolines and larger variations in EFS and EPD. These observations about the CFMAs are in line with previous studies [30, 31] and demonstrate the capability of the proposed procedure for applicator characterization with a diversity of setup conditions.

The use of a Cartesian robot offers superior adaptability to phantom and applicator geometries as well as specific requirements for QA procedures. Reference full-volume scans provide detailed 3D SAR distributions, and they can establish baseline data, for instance with a larger variation in relevant setup conditions (bolus thickness, curvature). However, volume scans may be impractical for routine QA. As shown in Sect. “EPD determination” of the “Results”, measuring two planes, i.e., the xy-plane and either the yz- or the xz-plane, does provide sufficient information to characterize applicators based on EFS and EPD. Routine QA would then be sufficient with scans of the central SAR planes and one or a few setup conditions (Fig. 3). Comparison of routine scans with reference scans in terms of EFS and EPD then allows us to evaluate applicator performance over time or detect malfunctions. The expected variability in the metrics can be taken from Table 2, although an in-house analysis of other applicators or setup conditions is recommended. As seen in Sect. “Spatial resolution evaluation” of the “Results”, the choice of spatial resolution can also be decided based on the available time and level of detail required for a routine measurement.

Each E‑field probe used should always be calibrated individually, as described in Sect. “Sensor calibration” of “Materials and methods”, to account for the nonlinearity of the sensor’s response and compute relative SAR values from measured E‑fields. Calibration should be repeated each measurement day, and it should cover the entire range of measured values during scans. It is essential to also cover the low power/voltage range sufficiently, to account for nonlinearities (Fig. 4) that would otherwise lead to wrong SAR distributions and EFS/EPD values. Other E‑field sensors, such as optical field probes, could also be used in similar setups, as previously demonstrated for deep hyperthermia equipment [37].

Previous studies measured E‑fields for SAR characterization using phantoms filled with saline solution and a built-in fat-equivalent layer [15]. While a fat layer provides a smoother electrical boundary, it makes the setup less practical and versatile, especially when different curvature conditions are to be evaluated. For this reason, we excluded a fat layer from our setup and observed no significant issues in the SAR distributions due to the electrical interfaces between setup elements. Regarding the saline solution concentration used, some prior studies used 6 g/L salinity (σ ~ 1.1–1.2 S m^−1^ at 434 MHz) [15, 31]. In line with other studies [16, 37, 38] we opted for the more commonly used 2/3 muscle-conductivity saline solution (3 g/L; σ = 0.53 S m^−1^).

For larger CFMAs, air pockets between the water bolus and electrode plates can impact SAR patterns, especially in a flat setup [30], and the use of a thin foam layer between the bolus and electrodes was proposed and clinically introduced to create a uniform air distribution and improve repeatability. The use of a foam layer in daily clinical practice also improves the repeatability of SAR delivered to patients.

The appropriate curvature and bolus thickness for QA and clinical use differs across different applicator types. The advantage of using CFMAs is that their bendability allows measurements with different curvature conditions and bolus thicknesses (Figs. 7 and 8), showing that the presented methodology is suitable for a wide range of superficial applicators. When implementing this QA procedure for other applicators, the curvature of the contact surface with the phantom should match the (fixed) curvature of the applicator. It is also important to evaluate the required spatial resolution, repeatability and criteria for EPD determination (Sects. “EPD determination”, “Repeatability evaluation” and “QA Procedure implementation” of the “Results”), since these aspects might be influenced by the operating frequency, effect of water bolus, applicator size, curvature and other characteristics relevant for that specific applicator type. The QA workflow outlined in this work and summarized in Sect. “QA Procedure implementation” of “Materials and methods” (Fig. 3) provides a general guideline procedure for defining these aspects, which can easily be customized for specific applicators.

The ALBA ON 4000D applicators have similar microstrip antennas and are geometrically similar to the CFMAs, with fixed curvatures of radii 17.35 cm (beta, gamma, delta) and 25 cm (alpha). Furthermore, the delta applicator has the same geometry as the 3H CFMA, with an aperture size of 30 × 22 cm^2^. The ALBA applicators should be characterized using a curved contact surface. The effects of different bolus thicknesses found in the present results and in other publications [15, 30–32] on CFMAs can be extended to QA measurements on the ALBA ON 4000D system. This holds particularly for the recommendation to avoid excessive bolus thickness. The 915 MHz BSD-500 system has three sizes of rigid waveguides, with an integrated water bolus. These waveguides are also better characterized with a flat contact surface. Due to its flat design, the 434 MHz LCA [29] is better characterized with a flat contact surface. Previous studies have recommended avoiding excessive bolus thickness and emphasized that the bolus should extend at least 2.5 cm beyond the applicator aperture [39].

Our QA procedure allows for comprehensive SAR characterization and is helpful for evaluating the performance of applicators over time and spotting possible defects or malfunctions. As shown in Sect. “Applicator characterization” of the “Results”, the qualitative observations about the influence of bolus thickness and curvature in the SAR distributions of the CFMAs are the same as in prior studies. The numerical values for EFS and EPD, on the other hand, may vary depending on setup details like salinity, use of foam or fat layers and curvature. It is thus important to compare the SAR-based applicator metrics quantitatively with other measurements using the same setup.

Specific absorption rate characterization using E‑field measurements and TR-based QA are complementary, since they provide independent assessments using different equipment and different physical principles. Compared to TR, E‑field-based SAR characterization has a simpler setup and enables faster measurements by eliminating the long waiting time between scans needed for TR. Both techniques can be used as part of a QA program, where the E‑field measurements could provide the basis for more frequent testing of applicator performance given their simplicity and lower time demand. Temperature rise measurements could then be performed less often, giving an independent check of heating properties of the applicators.

The proposed workflow, performing automated measurements in a liquid phantom, is similar to the procedure for dosimetric characterization in radiotherapy. This is an advantage for implementation, since hyperthermia treatments are usually in combination with radiotherapy, and most hyperthermia departments are located within or near a radiation oncology department. Implementing a robust QA program is fundamental in radiotherapy to ensure the accuracy and safety of treatments, combining, for example, less frequent reference dosimetry with fast dose metric monitoring, ensuring independent control. A similar concept could be further developed in hyperthermia, with the combination of TR and E‑field measurements.

The QA procedure described here provides ample versatility for implementation with a diversity of RF/microwave hyperthermia equipment. Integration within a comprehensive hyperthermia QA program can help to ensure safety and efficacy of hyperthermia treatments.

## Conclusion

This work presents a fast and efficient QA procedure for SAR-based characterization of radiofrequency and microwave hyperthermia applicators, similar to the dosimetric characterization procedures in radiotherapy. Using a Cartesian robot to move E‑field sensors in a liquid saline phantom allowed for fast measurements of ~40 min per setup condition. Measurements were easily implemented and automated, showing accuracy and repeatability in the obtained metrics, as well as versatility regarding setup. Through the demonstration of the procedure with superficial hyperthermia CFMAs, general considerations were made regarding spatial resolution, repeatability and criteria for EPD determination. The procedure is widely applicable to other types of radiative hyperthermia applicators and can be easily incorporated into a comprehensive hyperthermia QA program.

## Data Availability

Research data are stored in an institutional repository and will be shared upon reasonable request to the corresponding author.
